# Insights into the Mechanism of Proliferation on the Special Microbes Mediated by Phenolic Acids in the *Radix pseudostellariae* Rhizosphere under Continuous Monoculture Regimes

**DOI:** 10.3389/fpls.2017.00659

**Published:** 2017-05-02

**Authors:** Hongmiao Wu, Junjian Xu, Juanying Wang, Xianjin Qin, Linkun Wu, Zhicheng Li, Sheng Lin, Weiwei Lin, Quan Zhu, Muhammad U. Khan, Wenxiong Lin

**Affiliations:** ^1^Fujian Provincial Key Laboratory of Agroecological Processing and Safety Monitoring, College of Life Sciences, Fujian Agriculture and Forestry UniversityFuzhou, China; ^2^Key Laboratory of Crop Ecology and Molecular Physiology, Fujian Agriculture and Forestry UniversityFuzhou, China; ^3^Key Laboratory of Ministry of Education for Genetics, Breeding and Multiple Utilization of Crops/College of Crop Science, Fujian Agriculture and Forestry UniversityFuzhou, China

**Keywords:** bacteria, microbe, monocropping, phenolic acid, rhizosphere

## Abstract

As potent allelochemicals, phenolic acids are believed to be associated with replanting disease and cause microflora shift and structural disorder in the rhizosphere soil of continuously monocultured *Radix pseudostellariae*. The transcriptome sequencing was used to reveal the mechanisms underlying the differential response of pathogenic bacterium *Kosakonia sacchari* and beneficial bacterium *Bacillus pumilus* on their interactions with phenolic acids, the main allelochemicals in root exudates of *R. pseudostellariae* in the monoculture system. The microbes were inoculated in the pots containing soil and the medicinal plant in this study. The results showed that the addition of beneficial *B. pumilus* to the 2-year planted soil significantly decreased the activity of soil urease, catalase, sucrase, and cellulase and increased the activity of chitinase compared with those in the 2nd-year monocropping rhizosphere soil without any treatment. However, opposite results were obtained when *K. sacchari* was added. Transcriptome analysis showed that vanillin enhanced glycolysis/gluconeogenesis, fatty acid biosynthesis, pentose phosphate, bacterial chemotaxis, flagellar assembly, and phosphotransferase system pathway in *K. sacchari*. However, protocatechuic acid, a metabolite produced by *K. sacchari* from vanillin, had negative effects on the citrate cycle and biosynthesis of novobiocin, phenylalanine, tyrosine, and tryptophan in *B. pumilus*. Concurrently, the protocatechuic acid decreased the biofilm formation of *B. pumilus*. These results unveiled the mechanisms how phenolic acids differentially mediate the shifts of microbial flora in rhizosphere soil, leading to the proliferation of pathogenic bacteria (i.e., *K. sacchari*) and the attenuation of beneficial bacteria (i.e., *B. pumilus*) under the monocropping system of *R. pseudostellariae*.

## Introduction

*Radix pseudostellariae* L. belongs to the Caryophyllaceae family. It is a common and popular medicine in China. It is mainly produced in Fujian Province in the southeast of China. *R. pseudostellariae* contains ginseng saponins, polysaccharides, amino acids, flavonoids, and cyclic peptides, which can be used as a cure for spleen deficiency, anorexia, and palpitations (Zhao W.O. et al., 2015). The typical average annual yield of *R. pseudostellariae* is about 5000 tons, accounting for more than 22 million dollars per year. However, this medicinal plant is also affected by the replanting disease, in which the consecutive monoculture of *R. pseudostellariae* leads to a serious decline in the biomass and quality of underground tubers. Consecutive monoculture of *R. pseudostellariae* caused the yield reduction by 33.3%, the polysaccharide content and ginseng saponins Rb1 of the tuberous root were reduced by 88.08 and 44.33%, respectively (Zeng et al., 2012). Zhang and Lin (2009) and Wu et al. (2016) reported that more than 70% of medicinal plants were attacked by the replanting diseases, leading to serious and negative influences on both the soil and the plant health. Moreover, farmers have increased the amount of pesticides and fertilizers to find the solution to these problems, at the cost of excessive pesticide residues, more capital investment, soil degradation, and environmental pollution. Therefore, it has become a top priority to elucidate the underlying mechanisms of replanting disease, especially in the case of medicinal plant production.

Root exudates are perceived as chemical signals of communication between roots and microorganisms (Hirsch et al., 2003; Neal et al., 2012; Zhang et al., 2013; Yuan et al., 2015; Rugova et al., 2017; Vergani et al., 2017). A growing body of evidence suggests that the imbalanced microbial populations mediated by the root exudates play crucial roles in replanting disease (Wu et al., 2015). As one of the important root exudate, phenolic acids are involved in allelopathy and cause replanting disease (Bais et al., 2004, 2006; Kulmatiski et al., 2008; Liu et al., 2017). The phenolic acids are prone to change the soil microbial community and indirectly cause autotoxicity to the monocultured plants (Zhou and Wu, 2011; Zhou et al., 2012; Li et al., 2014; Liu et al., 2017). Previous results have also shown that consecutive monoculture of *R. pseudostellariae* can significantly increase the number of pathogenic microorganisms such as *Fusarium oxysporum*, *Talaromyces helicus*, *Kosakonia sacchari*, and so forth in the rhizosphere. Phenolic acids could promote the growth of these soil-borne pathogens (Zhao Y. et al., 2015; Wu et al., 2016). However, these phenolic acids did not show any autotoxicity toward *R. pseudostellariae.* Besides, previous studies demonstrated that *K. sacchari* produced protocatechuic acid when consuming phenolic acids and its metabolite, and protocatechuic acid negatively affected the growth of beneficial bacterium *Bacillus pumilus* (Wu et al., 2016). The pathogenic bacterium *K. sacchari* and the beneficial bacterium *B. pumilus* were isolated from the rhizosphere soil of the monocultured *R. pseudostellariae*. They are thought to be involved in the replanting disease of *R. pseudostellariae* (Wu et al., 2016). The studies also depicted that *B. pumilus* was not pathogenic to *R. pseudostellariae* but suppressed the mycelial growth of pathogens such as *F. oxysporum* and *T. helicus*. However, the intrinsic mechanism of the relationship between these special microbes and the root exudates is still not clarified.

Sequencing and analysis of expressed sequence tags have been widely used by most researchers for molecular marker discovery and gene expression profiling (Venturini et al., 2013; Mudalkar et al., 2014). The comparative transcriptome sequences were used in this study to identify the differentially expressed genes (DEG) in the bacteria under the treatment of phenolic acids in root exudates from the monocultured *R. pseudostellariae*. The influence of special microbes (*K. sacchari* and *B. pumilus*) on the physiological characteristics of *R. pseudostellariae* and its monoculture rhizosphere soil was also analyzed. This study focused on the underlying mechanisms related to the imbalance of microbial populations mediated by phenolic acids. It aimed to provide useful information and insights into the molecular ecological mechanism of destabilization of microbial populations mediated by phenolic acids present in secreted root exudates.

## Materials and Methods

### *R. pseudostellariae* Plant Preparation

The tubers of *R. pseudostellariae* were transplanted into pots (13-cm bottom diameter, 22-cm top diameter, and 20 cm height) containing different soils, which were sampled from the upper soil layer (0–15 cm) of the fields in the experimental station of Fujian Agriculture and Forestry University, Fuzhou, China. One of the soil samples was called as newly planted soil, which was never planted with *R. pseudostellariae* before, and the others were sampled from the field monocultured with *R. pseudostellariae* plants for 1 year. Each pot had four *R. pseudostellariae* plants. The young plants were grown in pots and incubated in the greenhouse.

The beneficial bacterium *B. pumilus* and the pathogenic bacterium *K. sacchari* of *R. pseudostellariae* were grown in Luria–Bertani (LB) medium at 37°C and 200 rpm. The specific bacterial cells were collected and washed three times with double-distilled water until an OD600 of 1 was reached, and then resuspended in the same double-distilled water (OD600 = 1). The bacterial cells were added to the two kinds of soils when *R. pseudostellariae* plants reached the five-leaf stage. In the sequential pot experiment, specific bacterial treatments were given to soils four times, which were called as newly planted soil and continuously monocultured soil, as mentioned earlier and in **Table 1**. The inoculum at the time of each treatment contained 60 mL of the bacterial cells in total. The pot experiment was conducted in three replicates. The details of the treatments are shown in **Table 1**.

**Table 1:** Different treatments of *Radix pseudostellariae.*

Treatments	Site code	January 19, 2016	April 1, 2016	April 12, 2016	April 18, 2016	April 25, 2016	April 29, 2016
Control (unplanted) soil	CK	Fallow	Fallow	Fallow	Fallow	Fallow	Sampled
Newly planted soil	FY	Planted					Sampled
One-year monoculture soil	SY	Planted					Sampled
One-year monoculture soil with *B. pumilus*	BP	Planted	The first treatment	The second treatment	The third treatment	The fourth treatment	Sampled
Newly planted soil with *K. sacchari*	KS	Planted	The first treatment	The second treatment	The third treatment	The fourth treatment	Sampled

*CK, unplanted soil; FY, first cropping year; SY, second cropping year; BP, Bacillus pumilus in the second cropping year; KS, Kosakonia sacchari in the first cropping year.*

### Physicochemical Properties of *R. pseudostellariae* and Rhizosphere Soil under the Treatments

#### Plant Chlorophyll Content and Soil Enzyme Activities

The soil urease activity was determined using the phenol–chloroform method and expressed as mg NH_3_⋅N g^-1^ soil 24 h^-1^. The cellulase activity was detected by the colorimetric anthracenone method and expressed as mg glucose g^-1^ soil 24 h^-1^. The sucrase activity was determined by the 3,5-dinitrosalicylic acid anthracenone method and expressed as mg glucose g^-1^ soil 24 h^-1^. The soil dehydrogenase activity was analyzed using the 2,3,5-triphenyltetrazolium chloride method on an oven-dried soil and expressed as mg TF g^-1^ soil 24 h^-1^. The chlorophyll content of *R. pseudostellariae* and the soil acid protease, chitinase, acid phosphatase, and catalase activities were determined using a plant and soil kit (Comin, Suzhou, China) according to the manufacturer’s protocol.

#### Phenolic Extraction and Determination

Soil phenolics were extracted as previously described (Zhou and Wu, 2011). Briefly, 5 g of soil was shaken in 25 mL of 1M NaOH for 24 h (200 rpm, 30°C) and then spun in a vortex generator for 30 min at maximum speed. After centrifugation at 10,000 rpm for 15 min, the supernatant was acidified to 2.5 with 9M HCl and extracted with ethyl acetate. The residue was dissolved in 5 mL of methanol using ultrasound for 5 min and maintained in the dark at 4°C.

The methanol solution of soil extracts was analyzed with the Waters HPLC system (C18 column: Inertsil ODS-SP, 4.6 × 250 mm, 5 μm). The mobile phase was a mixture of methanol and 2% acetic acid. Detection was performed at 280 nm. Protocatechuic acid and vanillin were identified and quantified by comparing the retention time and area with the pure standard.

### Transcriptome Analysis of Bacteria

#### Bacterial Cell and RNA Extraction

The LB liquid culture medium was diluted six times and sterilized for 20 min. The culture medium was cooled, an appropriate amount of each protocatechuic acid and vanillin, passed through a 0.22-μm ultrafiltration membrane, was added to make the concentration up to 120 μmol/L, which was the closest to actual conditions detected in the rhizosphere soil. The *K. sacchari* culture (50 μL), which had already been activated, was added to the vanillin tube and placed on a thermostatic shaker at 200 rpm for 6.5 h at 37°C. *B. pumilus* was added to protocatechuic acid tube and cultured for 7.5 h at 37°C. The control received only sterile water to substitute the effects of phenolics. Then, all the bacterial cells were centrifuged, washed twice with RNase-free water, and maintained at -80°C.

The total RNA was isolated from each tissue sample using the RNAiso Plus kit (TaKaRa, Dalian, China) according to the manufacturer’s protocol. The purity and content of each RNA were measured using the Qubit RNA Assay Kit in Qubit 2.0 Fluorometer (Life Technologies, Carlsbad, CA, USA) and confirmed using 1.2% agarose gels.

#### cDNA Library Construction and Sequencing

The probes were used to eliminate the rRNA sequence of prokaryotes. Then, the mRNA was concentrated using oligo (dT) magnetic adsorption and then broken into fragments, which were used as templates to synthesize first-and second-strand cDNA. The double-stranded cDNA was further purified using the QIAQuick Polymerase Chain Reaction (PCR) extraction kit (Qiagen, Hilden, Germany), resolved for final reparation and poly (A) addition, and then connected with different sequencing adaptors. The library was sequenced by Biomarker Technologies Co., Ltd. (Beijing, China) using the Illumina HiSeq 2500 system.

### Transcriptome Assembly, Gene Annotation and Expression, and Gene Ontology and Kyoto Encyclopedia of Genes and Genomes Annotation

The raw sequencing data reads were first cleaned by removing adaptor sequences, poly-N and low-quality reads. All the downstream analyses were based on high-quality data. For *de novo* assembly, the clean reads were mapped back to the contigs by Trinity (Grabherr et al., 2011) with the parameters set at a similarity of 90%. Subsequently, the contigs were assembled to construct transcripts with pair-end information and clustered to obtain unigenes.

A sequence similarity search was performed against several databases to investigate the putative functions of the unigenes based on sequence or domain alignment. All unigenes were compared with genes in the non-redundant (NR) databases (National Center for Biotechnology Information non-redundant protein^1^), Swiss-Prot^2^, Kyoto Encyclopedia of Genes and Genomes (KEGG^3^), Clusters of Orthologous Groups (COG^4^), and Gene Ontology (GO^5^) (Ashburner et al., 2000; Tatusov et al., 2000; Kanehisa et al., 2004). Homology search against the NR database was performed to identify top-hit species by BLASTX with a cut-off *E*-value < 10^-5^. The Blast2GO program (Conesa et al., 2005) was used to obtain the functional classification, and the WEGO software (Ye et al., 2006) was employed to perform the distribution of GO functional classification.

### Quantitative Real-Time PCR Analysis

The expression analysis of the selected genes was assessed using quantitative real-time PCR (qRT-PCR). The reaction conditions were as follows: 95°C for 15 min, followed by 40 cycles of 95°C for 50 s, 55°C for 40 s, and 72°C for 40 s. At least four biologically independent replicates were used for each sample. All the primer sequences involved in the experiment used for qRT-PCR are listed in Supplementary Tables S1, S2.

### Biofilm Formation Assay

An amendment assay was performed to determine the effects of the phenolic acids on bacterial biofilm formation, as described by previous reports in 96-well microtiter plates (Hamon and Lazazzera, 2001). In short, protocatechuic acid and vanillin, with concentration up to 120 μmol/L, were added to the culture medium and incubated at 37°C for 36 h. Then, the biomass of the biofilms was harvested and stained with 250 μL of 0.1% crystal violet. The bound crystal violet was further solubilized with 250 μL of 4:1 (v:v) ethanol and acetone. The multifunctional plate reader SpectraMax i3 analysis system (Multi-Mode Detection Platform, USA) was used to measure the OD570 of the solution in each well.

### Statistical Analysis

The GO annotation was analyzed using the Blast2GO software^6^. Functional classification of the unigenes was performed using the WEGO software. Differences among the treatments were calculated and statistically analyzed using the analysis of variance and the least significant difference multiple range test (*P* < 0.05). The Statistical Package for the GraphPad Prism version 5.1 and the Data Processing System version 7.05 were used for statistical analysis.

## Results

### Physicochemical Properties of *R. pseudostellariae* and Rhizosphere Soil under the Treatments

**FIGURE 1:**
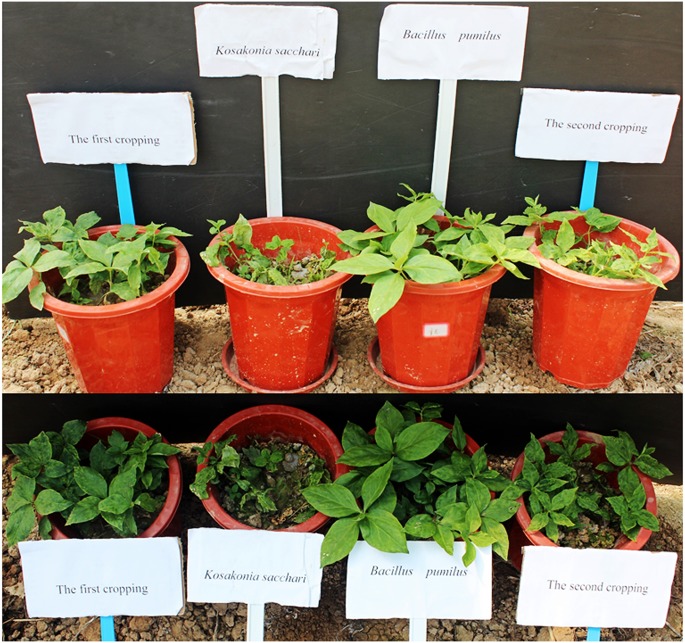
**Photographs of *Radix pseudostellariae* under different treatments**.

**Figure 1** shows that *B. pumilus* had a better effect on the growth of *R. pseudostellariae* in the soil in the second cropping year. *B. pumilus* significantly promoted the chlorophyll content and plant biomass of *R. pseudostellariae*, while the opposite was true in the case of pathogenic bacterium *K. sacchari*, as shown in Supplementary Figure S1.

**FIGURE 2:**
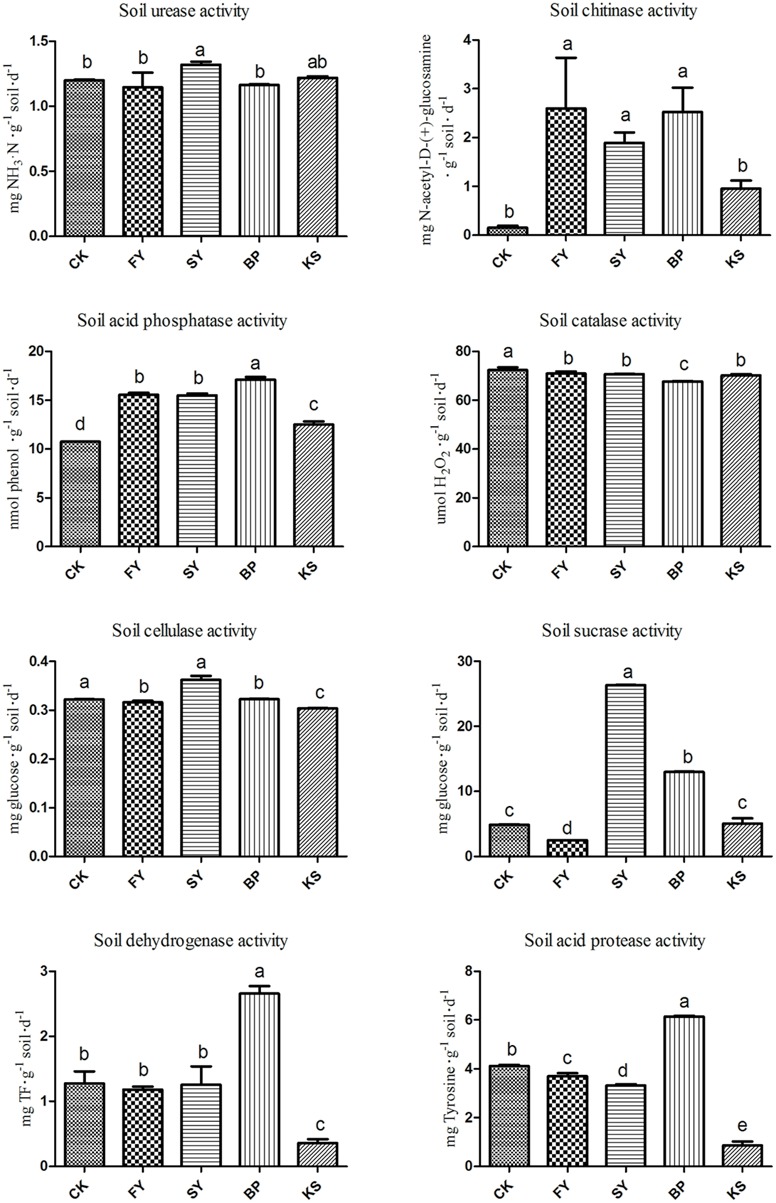
**Soil enzyme activities in the rhizosphere soil of *Radix pseudostellariae* under different treatments.** FY represents the first cropping year, SY the second cropping year, BP the treatment of *Bacillus pumilus* in the second cropping year, and KS the treatment of *Kosakonia sacchari* in the first cropping year. Columns with different letters are statistically different (LSD-test, *p* < 0.05).

The activity of soil chitinase and acid protease decreased, and the activity of soil urease, cellulase, and sucrase significantly increased in the soil as it was continuously monocultured for 2 years. The added *B. pumilus* significantly increased the activity of soil acid phosphatase, dehydrogenase, and acid protease compared with enzyme activities in the soil monocultured for 2 years without the beneficial bacterial treatment. However, the added pathogenic *K. sacchari* significantly decreased the activity of soil chitinase, acid phosphatase, cellulase, dehydrogenase, and acid protease (**Figure 2**) compared with the enzyme activities in the newly planted soil without the bacterial addition.

The horizontal axis is the number of the unigenes and proportion to the total number, and the vertical axis is the name of KEGG metabolic pathway.

HPLC analysis showed that the protocatechuic acid levels tended to increase in the rhizosphere soil as the number of continuous monoculture years increased. It was also found that the vanillin tended to decrease, but protocatechuic acid increased in the newly planted soil treated with the pathogenic *K. sacchari*, compared with the phenolic acids in the newly planted soils without the pathogen treatment (Supplementary Figures S2, S3).

### *De Novo* Assembly of Transcriptome

The transcriptome of bacteria was characterized, and a number of genes associated with functional regulation and metabolism were obtained. Sequencing of the bacterial transcriptome yielded about 21,797,093 clean reads with 93.62% Q30 bases and 5.48 Gb with 43.16% GC ratio in *B. pumilus*. The 24,161,020 clean reads with 93.55% Q30 bases and 6.08 Gb with 54.06% GC ratio were obtained in *K. sacchari* (**Table 2**). Transcripts were assembled into unigenes, yielding 131 DEG with downregulation of 77 in *B. pumilus*. The number of DEG detected in *K. sacchari* was up to 331, including 235 upregulated unigenes (**Table 3**).

**Table 2:** Summary of data generated in the transcriptome sequence of bacteria.

Transcriptome sequencing results	BPCK	BP	SMCK2	SM2
Clean reads	11,703,897	10,093,196	11,801,236	12,359,784
Clean bases	2,942,889,574	2,540,058,366	2,971,006,882	3,111,260,768
Q30 (%)	94.45	92.79	93.84	93.25
GC (%)	43.13	43.19	54.16	53.96

**Table 3:** Statistics of differentially expressed genes under the bacterial treatments.

Type	COG	GO	KEGG	Swiss-Prot	NR	Total	Pfam	Upregulated	Downregulated
BPCK_vs_BP	94	76	94	115	131	131	–	54	77
SMCK2_vs_SM2	242	218	216	297	331	–	307	235	97

*COG, clusters of orthologous groups; GO, gene ontology; KEGG, kyoto encyclopedia of genes and genomes; NR, non-redundant.*

### COG Annotation

**FIGURE 3:**
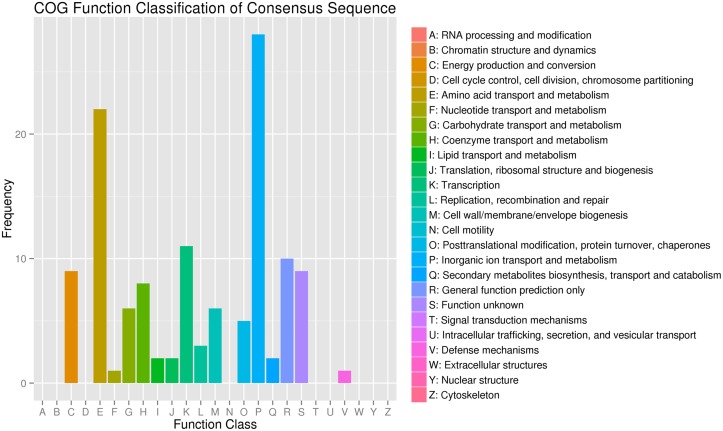
**Clusters of orthologous groups of proteins annotated classification for the unigenes between the treatment (BP) and control (BPCK) in *B. pumilus***.

To assess the validity and integrity of the transcriptome sequence in *B. pumilus*, 131 DEG annotated in the NR database were assigned to the COG database to classify potential functions. In total, 125 DEG were aligned to the 16 COG classifications. Among them, assignments to (P) inorganic ion transport and metabolism made up the majority (28, 22.4%), followed by (E) amino acid transport and metabolism (22, 17.6%), (K) transcription (11, 8.8%), and (R) general function prediction only (10, 8%) (**Figure 3**).

**FIGURE 4:**
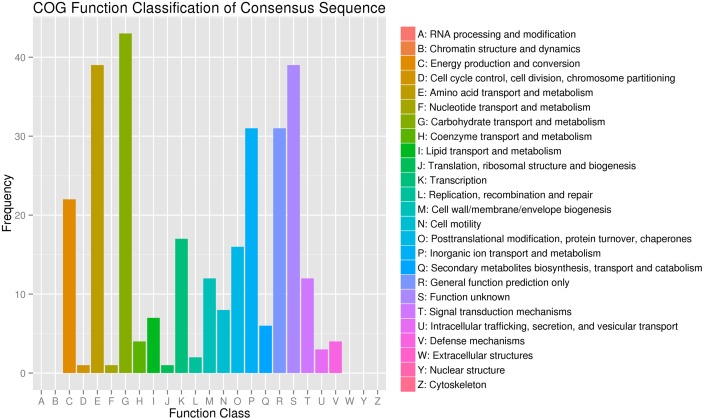
**Clusters of orthologous groups of proteins annotated classification for the unigenes between the treatment (SM2) and control (SMCK2) in *K. sacchari***.

A total of 299 DEG in *K. sacchari* were aligned to the 20 COG classifications. The majority part was (G) carbohydrate transport and metabolism (43, 14.38%), followed by (E) amino acid transport and metabolism (39, 13.04%), (S) function unknown (39, 13.04%) and (P) inorganic ion transport and metabolism (31, 10.37%), (R) general function prediction only (31, 10.37%), and (C) energy production and conversion (22, 7.36%) (**Figure 4**).

### GO Annotation of Gardenia Transcriptome

**FIGURE 5:**
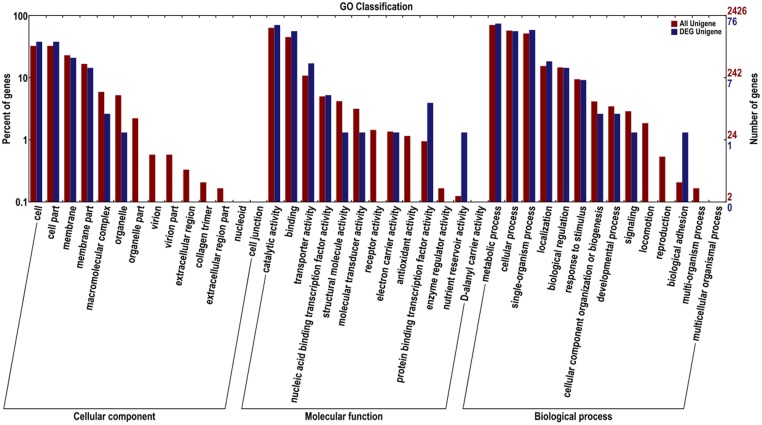
**Gene ontology for the unigenes and DEG between sample treatment (BP) and control (BPCK) in *B. pumilus***.

The Blast2GO software was used to assemble the DEG in *B. pumilus*. These DEG were assigned to 25 classifications. In the category of cellular component, most genes were associated with cell, cell part, membrane, membrane part, macromolecular complex, and organelle. In the category of molecular function, genes belonged to catalytic activity, binding, transporter activity, protein binding transcription factor activity, and nutrient reservoir activity. In the category of biological process, the dominant subcategories were genes associated with the metabolic process, single-organism process, cellular process, localization, biological regulation, stimulatory response, cellular component organization or biogenesis, developmental process, biological adhesion, and signaling (**Figure 5**).

**FIGURE 6:**
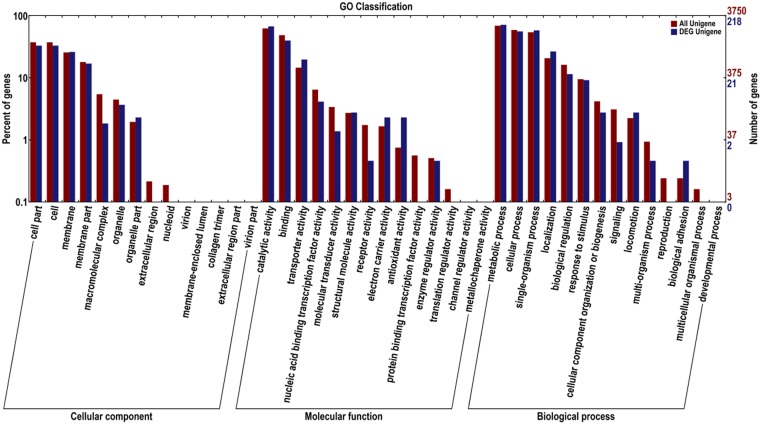
**Gene ontology for the unigenes and DEG between sample treatment (SM2) and control (SMCK2) in *K. sacchari***.

The DEG in *K. sacchari* were assigned to 28 classifications. Among the category of cellular component, the dominant subcategories were genes associated with cell, cell part, membrane, membrane part, and organelle. The category of molecular function included most of the genes associated with catalytic activity, binding, transporter activity, nucleic binding transcription factor activity, structural, molecule activity, electron carrier activity, antioxidant activity, and molecular transducer activity. The metabolic process, single-organism process, cellular process, localization, biological regulation, stimulatory response, locomotion, and cellular component organization or biogenesis were under the category of biological process (**Figure 6**).

### KEGG Pathway Annotation

**FIGURE 7:**
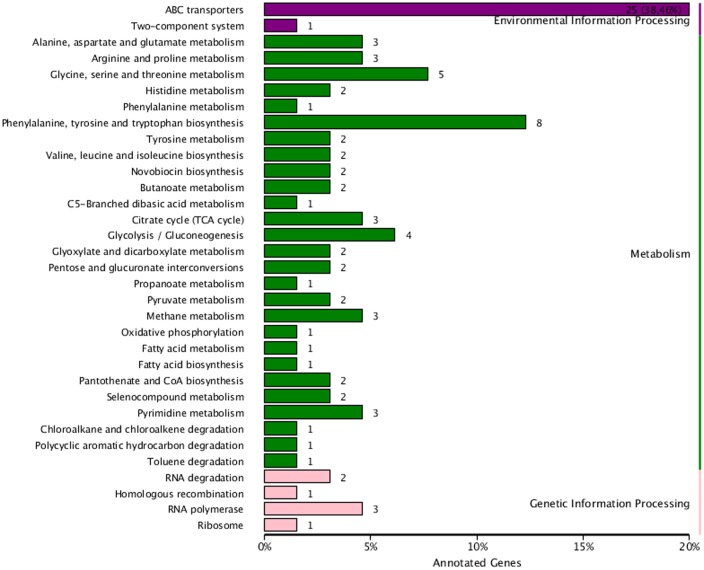
**Kyoto encyclopedia of genes and genomes classification chart for the DEG between sample treatment (BP) and control (BPCK) in *B. pumilus***.

The KEGG database was used to understand the interaction of genes and metabolic biological functions in *B. pumilus*. The 94 DEG were assigned to 33 KEGG pathways. The three KEGG categories were based on pathway data, including metabolism (61, 64.89%), environmental information processing (26, 27.66%), and genetic information processing (7, 7.45%). Among the pathway subgroups, the pathway of ABC transporters had the most unigenes (25), followed by phenylalanine, tyrosine, and tryptophan biosynthesis (8), and glycine, serine, and threonine metabolism (5). The metabolism pathways of citrate cycle (3), fatty acid biosynthesis (1), arginine and proline metabolism (3), histidine metabolism (2), and novobiocin biosynthesis (2) also had several DEG (**Figure 7**).

**FIGURE 8:**
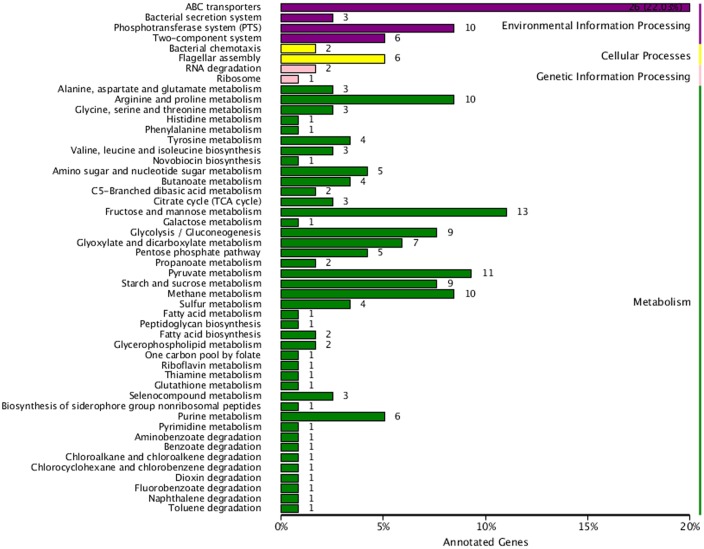
**Kyoto encyclopedia of genes and genomes classification chart for the DEG between sample treatment (SM2) and control (SMCK2) in *K. sacchari*.** The horizontal axis is the number of the unigenes and proportion to the total number, and the vertical axis is the name of the KEGG metabolic pathway.

A total of 201 DEG were assigned to 55 KEGG pathways, including metabolism (145, 72.14%), environmental information processing (45, 22.39%), cellular processing (8, 3.98%), and genetic information processing (3, 1.49%) in *K. sacchari.* The pathways of ABC transporters (26), fructose and mannose metabolism (13), pyruvate metabolism (11), phosphotransferase system (PTS; 10), and glycolysis/gluconeogenesis (9) were the dominant unigenes. The ratio of the four classifications showed that some DEG were focused on the flagellar assembly (6), fatty acid biosynthesis (2), bacterial chemotaxis (2), and phenylalanine, tyrosine, and tryptophan biosynthesis (1) (**Figure 8**).

### Validation of Expression of Selected DEG Using qRT-PCR

**FIGURE 9:**
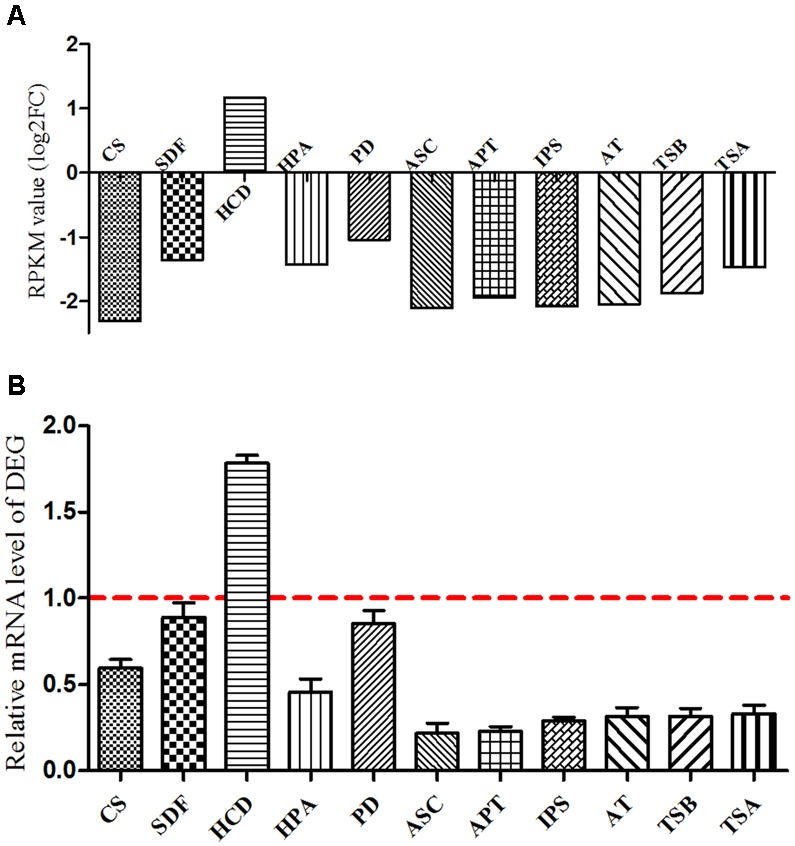
**Verification of differentially expressed genes between sample treatment (BP) and control (BPCK) in *B. pumilus*. (A)** Represents the RPKM value; **(B)** represents the differentially expressed genes determined by qRT-PCR. CS, citrate synthase; SDF, succinate dehydrogenase flavoprotein subunit; HCD, 3-hydroxyacyl-CoA dehydrogenase; HPA, histidinol-phosphate aminotransferase; PD, prephenate dehydrogenase; ASC, anthranilate synthase component I; APT:IPS, indole-3-glycerol phosphate synthase; AT, N-(5′-phosphoribosyl) anthranilate isomerase; TSB, tryptophan synthase subunit beta; TSA, tryptophan synthase subunit alpha.

Based on the KEGG pathway enrichment scatter diagram (Supplementary Figure S4), the expression of downregulated DEG involved in the important metabolic pathways (citrate cycle; fatty acid metabolism; phenylalanine, tyrosine, and tryptophan biosynthesis; and novobiocin biosynthesis) were determined by real-time PCR in *B. pumilus*. The results showed that the trend of these unigenes was similar to the RPKM values. Based on the DEG and metabolic pathway, it was concluded that the protocatechuic acid had a negative effect on the growth and biocontrol of *B. pumilus* (**Figure 9**).

**FIGURE 10:**
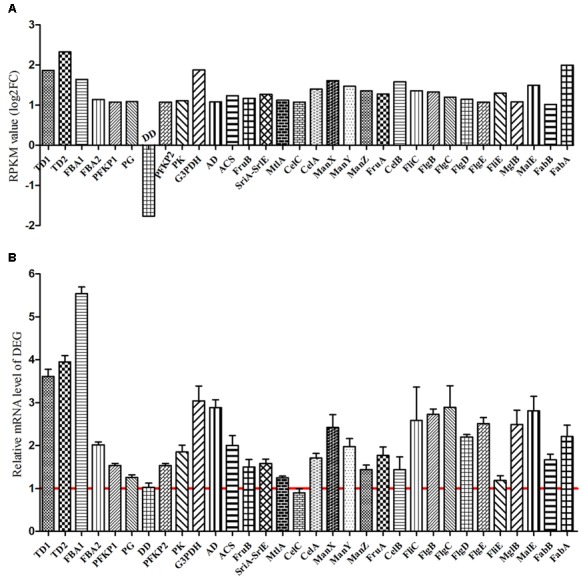
**Verification of differentially expressed genes between sample treatment (SM2) and control (SMCK2) in *K. sacchari*. (A)** Represent the RPKM value; **(B)** represent the determined differentially expressed genes by qRT-PCR. TD1, transketolase; TD2, transaldolase; FBA1, fructose-bisphosphate aldolase; FBA2, fructose-bisphosphate aldolase; PFKP1, 6-phosphofructokinase; PG, phosphoglyceromutase; DD, dihydrolipoamide dehydrogenase, partial; PFKP2, 6-phosphofructokinase; PK, pyruvate kinase; G3PDH, glyceraldehyde-3-phosphate dehydrogenase; AD, glyceraldehyde-3-phosphate dehydrogenase; ACS, acetyl-CoA synthetase; FruB, PTS fructose transporter subunit IIA; SriA-SrlE, PTS sorbitol transporter subunit IIC; MtlA, PTS mannitol transporter subunit IIABC; CelC, PTS lactose transporter subunit IIA; CelA, PTS lactose transporter subunit IIB; ManX, PTS system sorbose subfamily IIB component; ManY, PTS mannose transporter subunit IIC; ManZ: PTS mannose transporter subunit IID; FruA, PTS fructose transporter subunit IIBC; CelB: cytochrome C biogenesis protein CcmF; FliC, flagellin; FlgB, flagellar biosynthesis protein FlgB; FlgC, flagellar basal body rod protein FlgC; FlgD, flagellar basal body rod modification protein; FlgE, flagellar hook protein FlgE; FliE, flagellar hook-basal body protein FliE; MglB, methyl-galactoside ABC transporter substrate-binding protein; MalE, sugar ABC transporter substrate-binding protein; FabB, 3-oxoacyl-ACP synthase; FabA, 3-hydroxydecanoyl-ACP dehydratase.

For *K. sacchari*, the KEGG pathway enrichment of the upregulated DEG in glycolysis/gluconeogenesis, fatty acid biosynthesis, pentose phosphate, bacterial chemotaxis, flagellar assembly, and PTS pathway was also determined by qRT-PCR (Supplementary Figure S5). Vanillin enhanced these pathways, having a positive effect on the growth of the pathogenic *K. sacchari* (**Figure 10**).

### Effects of Phenolic Acids on the Biofilm Formation of Specific Bacteria

The results showed that vanillin significantly stimulated the biofilm formation of the pathogenic bacterium *K. sacchari* at a dose of 120 μmol/L, while protocatechuic acid showed inhibitory effects on the beneficial bacterium *B. pumilus* (Supplementary Figure S6).

## Discussion

Replanting disease is a common example of typical negative plant–soil feedback, which accounts for the serious decline in the biomass and quality of crops when the same crop or its related species are monocultured consecutively in the same soil (Huang et al., 2013). Replanting disease has become a prevalent problem recently in the production of many annual crops, which are being subjected to intensive consecutive monoculture, such as *Rehmannia glutinosa*, cucumber, peanut, and tobacco (Zhou et al., 2012; Li et al., 2014; Santhanam et al., 2015; Wu et al., 2015). Many factors have been thought to be responsible for the replanting disease, including soil nutrient imbalance, soil physical and chemical properties, accumulation of autotoxins generated by roots, and change in the soil microbial community structure (Wu et al., 2011; Huang et al., 2013; Zhao et al., 2016). With more research focused on this field, many studies have revealed that root exudates produced by plants have a propensity to shape the rhizosphere microbiome directly or indirectly and also have some influences on the growth of plants (Li et al., 2014; Venturelli et al., 2015; Wu et al., 2016). A previous study found that the root exudates of *R. glutinosa* could promote the growth and toxin production of pathogenic *F. oxysporum* while inhibiting the growth of the beneficial bacteria *Pseudomonas* sp (Wu et al., 2015). Liu et al. (2017) have elucidated the benzoic acid significantly increased the abundances of bacterial and fungal, and reduced bacteria-to-fungi ratio in the rhizosphere soil of peanut. Moreover, the continuous monoculture of *R. pseudostellariae* was found to differentially mediate the shifts in rhizosphere microbes, showing that the number of the pathogenic bacterium *K. sacchari* in the rhizosphere soil of *R. pseudostellariae* significantly increased with the increase in monocropping years, and the opposite trend was observed in the case of *B. pumilus* (Wu et al., 2016). This study demonstrated the positive effect of *B. pumilus* on the growth of *R. pseudostellariae*, suggesting that the addition of beneficial bacterium *B. pumilus* to the monocultured rhizosphere soil of the medicinal plants significantly decreased the activity of soil urease, catalase, sucrase, and cellulase and increased the activity of chitinase compared with those in the 2nd year monocultured soil without the treatment. These changes could contribute to the better growth of the plants and inhibit the proliferation of pathogenic fungi (Mauch et al., 1988; Zeng et al., 2007). However, the added *K. sacchari* shifted most soil enzyme activities, creating a disease-conducive environment to inhibit the growth of *R. pseudostellariae*. The results further confirmed the imbalance of microorganism population structure, which in turn changed the properties of rhizosphere biology including the amounts and activities of soil enzymes by mediating the phenolic acid exudates under the cropping system.

**FIGURE 11:**
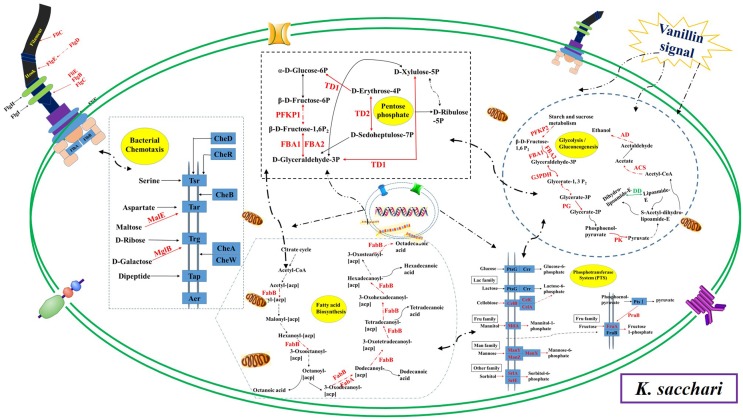
**Schematic presentation of some biological pathways affected in *K. sacchari* under vanillin stress.** Most differentially expressed genes were integrated and are indicated in red (upregulated) or green (downregulated), respectively.

**FIGURE 12:**
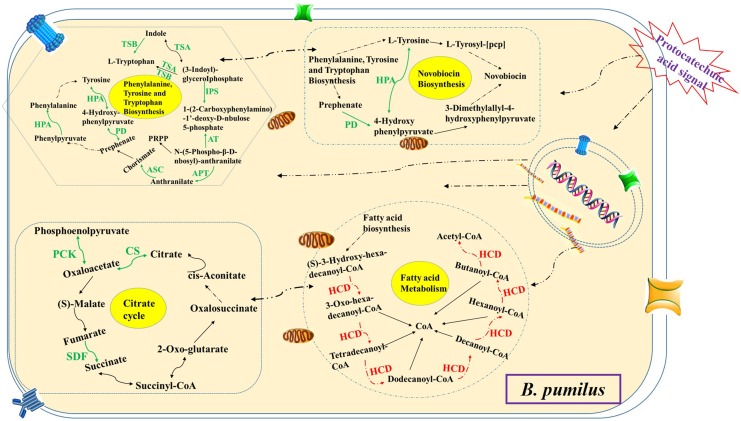
**Schematic presentation of some biological pathways affected in *B. pumilus* under protocatechuic acid stress.** Most differentially expressed genes were integrated and are indicated in red (upregulated) or green (downregulated), respectively.

The microbes in the rhizosphere, referred to as the second genome of the plants, and the plants are able to shape their rhizosphere microbiome by hosting specific microbial communities (Berendsen et al., 2012). Zhou and Wu (2011) and Zhou et al. (2012) reported that the root exudates of cucumber could change soil microbial communities and promote the growth of *F. oxysporum* f.sp. *cucumerinum* (the host-specific soil-borne pathogen of cucumber) in the continuously monocropped system. For *R. pseudostellariae*, *K. sacchari* and *B. pumilus* have been identified as the specific microbial flora involved in the replanting disease (Wu et al., 2016). However, the rhizosphere is a complex system. The catabolism of the root exudates and their stimulatory effects on the microbial community structure has been explored by studying the effects of single low-molecular-weight organic molecules in simple systems (Landi et al., 2006; Renella et al., 2007; Li et al., 2014). This study found vanillin as one of the main allelochemicals in the root exudates of the medicinal plants, which enhanced the glycolysis/gluconeogenesis, and pentose phosphate, and PTS pathway, leading to high metabolism and a good balance between carbon and nitrogen in the treated pathogenic *K. sacchari*. Moreover, the genes associated with the fatty acid biosynthesis, flagellar assembly, and bacterial chemotaxis were upregulated in the pathogenic bacteria under treatment (**Figure 11**). Chemotaxis and colonization are the two primary elements of plant–microbe interactions. Moreover, bacterial biofilm formation on plant roots is a visualized performance of effective colonization (Sood, 2003; Timmusk et al., 2005; Li et al., 2013). It means that vanillin promotes colonization and growth of the treated *K. sacchari* and leads to an increased incidence of the disease. Moreover, the metabolite (protocatechuic acid) of *K. sacchari* had a negative effect on the citrate cycle, novobiocin biosynthesis, and phenylalanine, tyrosine, and tryptophan biosynthesis of the beneficial *B. pumilus* (**Figure 12**). It also promoted the fatty acid metabolism and inhibited the biofilm formation of *B. pumilus* under treatment. The results suggested that the metabolite (protocatechuic acid) of *K. sacchari* in the rhizosphere soil was not conducive to the colonization of *B. pumilus* and significantly reduced its antagonistic ability against the specific pathogens in the monoculture system.

The complex plant-associated microbial communities are crucial for plant health. However, the microbial activity in soil is greatly influenced by plant root exudation, resulting in an increased or decreased microbial biomass and activity around the roots (Bais et al., 2006; Doornbos et al., 2012). For most plant species, consecutive monoculture creates a new environment for the build-up of specialized plant pathogens (Bennett et al., 2012). In this process, the root exudates of plants play important roles. Phenolic acids act as signals to attract *K. sacchari* by chemotaxis response. *K. sacchari* utilized vanillin for its proliferation. The stimulation of vanillin caused high metabolism and maintained a good balance between carbon and nitrogen in *K. sacchari*. The colonization of *K. sacchari* could be promoted by the biofilm formation. *K. sacchari* altered the soil enzyme activities and caused the disease in *R. pseudostellariae*. Meanwhile, the metabolite of *K. sacchari* had a negative effect on the growth of beneficial bacteria. Wang et al. (2013) reported that *Pseudomonas* utilized and catalyzed the conversion of (–)-catechin into protocatechuic acid, which then became an active agent in the allelopathy of understory species in the rhizosphere of *Rhododendron formosanum*. The results showed that the pathogenic bacterium *K. sacchari* was able to convert vanillin, one of the main allelochemicals in root exudates of *R. pseudostellariae*, into protocatechuic acid, and reduced the number of beneficial bacteria via chemical transformation in the monoculture cropping of *R. pseudostellariae*. The function of the root exudates differs in plant species and their rhizosphere microbes. The present study unraveled the molecular mechanism underlying differential changes in specific hosting microflora mediated by the root exudates of *R. pseudostellariae* in the monoculture system. The results gave a promising strategy for the control of replanting disease by rhizosphere management, which includes root exudate remediation, organic amendment application, and rhizosphere microbiome manipulation. In short, rhizosphere management is bound to lead to a new round of the Green Revolution, which will have a huge influence on the sustainability development of agriculture.

## Author Contributions

WXL and HW conceived the study. HMW, WXL, and MK wrote the paper. HMW, XQ, QZ, JW, and ZL performed experiments. HMW, LW, and SL performed the statistical analyses. HMW, SL, and WWL are involved in field management and soil sampling. MK, JW, and JX have revised the manuscript. All authors discussed the results and commented on the manuscript.

## Conflict of Interest Statement

The authors declare that the research was conducted in the absence of any commercial or financial relationships that could be construed as a potential conflict of interest.

## Footnotes

1 http://www.ncbi.nlm.nih.gov

2 http://www.expasy.ch/sprot

3 http://www.genome.jp/kegg

4 http://www.ncbi.nlm.nih.gov/COG

5 http://www.geneontology.org/

6 https://www.blast2go.com/
