# Supplementary material for: Insights into the Mechanism of Proliferation on the Special Microbes Mediated by Phenolic Acids in the Radix pseudostellariae Rhizosphere under Continuous Monoculture Regimes
Source: Front Plant Sci. 2017 May 2;8:659. doi: 10.3389/fpls.2017.00659 (PMC5411758; doi:10.3389/fpls.2017.00659)
Supplement: Supplementary file 1 [file Data_Sheet_1.PDF]

Insights into the mechanism of proliferation on the special microbes mediated by phenolic acids in the *Radix pseudostellariae* rhizosphere under continuous monoculture regimes

Hongmiao Wu[1,2†], Junjian Xu[1,2†],Juanying Wang[1,2], Xianjin Qin[2,3], Linkun Wu[1,2], Zhicheng Li[1,2], Sheng Lin[1,2], Weiwei Lin[1,2], Quan Zhu[1,2], Muhammad Umar Khan[1,2], Wenxiong Lin[1,2,3*]

[1] Fujian Provincial Key Laboratory of Agroecological Processing and Safety Monitoring, College of Life Sciences, Fujian Agriculture and Forestry University, Fuzhou 350002, PR China.

[2] Key Laboratory of Crop Ecology and Molecular Physiology (Fujian Agriculture and Forestry University), Fujian Province University, Fuzhou 350002, PR China.

[3] Key Laboratory for Genetics, Breeding and Multiple Utilization of Crops, Ministry of Education / College of Crop Science, Fujian Agriculture and Forestry University, Fuzhou 350002, PR China.

.

Corresponding author:

Prof. Wenxiong Lin

College of Life Sciences, Fujian Agriculture and Forestry University;

Fuzhou 350002, Fujian, People's Republic of China;

E-mail: wenxiong181@163.com

[†]These authors have contributed equally to this work.

Supplemental Table S1 PCR primers used in qRT-PCR of *Bacillus pumilus.*

Target genes	Forward primer (5`-3`)	Reverse primer (5`-3`)
APT	GGAGATGATGGGCTGGATG	CTGGTGATTGGACCTGAATG
CS	AAATGCAGCGACAAGTCCAG	TATCCTTATTAGGTTTGCTTGATGA
SDF	GCATCCAGCCTCTTGTAAACAC	GCAAACTGCGGGAACTCT
HCD	AAAATGCCGAAGGAGACC	CGAGATTCGCACCAACAC
HPA	TAAGAACGGAACTGGCTGAG	GGAATGACGGAGATGGGAT
PD	GCAGGATCTCATAAGTCAGGTG	GCATCATGTTCTTCGGGTGT
ASC	ATGATGTGGGGCGTGTAGC	TGGAACGGAATCAGCGAC
IPS	GAAGCCCTTATGAATCCTCACC	ATGGCATCTGCCCCTATCC
AT	ACGGCATTCGCTCCAAGT	GGTGCAAGTTTTCCCCAGTT
TSB	CGAAACAGGAGCAGGTCAG	ATACGGATGAGGTCCAACG
TSA	GCACCAACAAGCAAGGAAC	CCGACAACGACACCATCAC
HPA	TAAGAACGGAACTGGCTGAG	GGAATGACGGAGATGGGAT

Supplemental Table S2 PCR primers used in qRT-PCR of *Kosakonia sacchari*

Target genes	Forward primer (5`-3`)	Reverse primer (5`-3`)
TD1	TCCGTTCCAACACCTTCG	CTTTCGCCTCCACCACTT
TD2	TTGTTGGCTTGAACTGCG	GGTGTCTTTGCTGGGGATA
FBA1	AACCACAACGAAACGCTCAG	TACGCAGGTACGCCCACAAG
FBA2	AACATAACCTGCCGCACAAC	AAGCCTGCTCCAGACGAGTCAC
PFKP1	GGCGTGGATGCCAATAAT	GAGTCCCCTGGTTGATGGT
PG	CTTGAACTGAACATCCCGACTG	CGCTTTACCCTGGTTTGCTAC
DD	GGCTCGGTGTAGGCGATAGA	AAAGCAGGCGTGGAAGTGG
PFKP2	GGCGTGGATGCCAATAAT	GAGTCCCCTGGTTGATGGT
PK	GCAGCCTCCTTCATCCGTA	GAATCGAGCATCTGGGTC
G3PDH	GTGCCGCCGCAGAAAACAT	CCGTATTCGTTGTCATACCAGGAG
AD	AAGCCCAGCGTGAATATG	AATCGTGCCGAAGGTGTC
ACS	TTTTCGGTGGTTTCTCGC	TTGTCGGTCAGCTCGTTCC
FruB	CTCTGGAGCGAAGGACAGGT	GTGCGGCTACGGAATCAT
SriA SrlE	CAGAAAGGCGGCGAAGTGT	GCAGTAAATAACGGGCAAGTGG
MtlA	AAAACTGACCCCGACCTATC	TTCACCGAAGCGGACACCT
CelC	ATTCAAACCCAGCTTATCG	ATCATATCGGTTGCCAGA
CelA	CAGCAGGTATGTCCACCA	ATGTCGATAACCGCCACT
ManX	CTACCCGCTGGACAAAAGA	ATAAGCCATGCCGCCAAT
ManY	ATTGCTCTGGGCTGGATG	AAAGTGCCGTCAGGTTGC
ManZ	ATATGGGCGGTGGTTTCC	GCACGGTCGTGACGTTAGTT
FruA	TCAACAAAGCGGCATACG	TACAGCACGGCAGAACACG
CelB	GCACATTGCCAGTGGATAGC	TCAGCGGATAAAGGGTCAG
FliC	AGTCAATCTCGGAGGGTA	CGTTGTAGTTCAGCGTCT
FlgB	GGACCGTCTACCGATTTGTT	GCTTTTGATCTGCCCACC
FlgC	GGCGGTGTAAAAGTTGCTG	AGCGTGAGGGTTTTGAGC
FlgD	AACAACACCAGCACAACCG	TTTTCAATCCCGCTCACG
FlgE	TTCGCCGATATGTTTGCC	TGCGGTTCTCATCCAGTTTG

FliE	AGGGCATTGAAAGCGTCG	GCTGCGTTTTGCGTATCG
MglB	TGACTCTGTCTGCTGTTATGGC	GGTCGTTCTGTTTGGATTGG
MalE	TGCCTACCCGATTGCTGT	TTCACGAGGAAACCGAGAC
FabB	TTCGTTATCGCAGGTGGC	CCGTGGGAGTTCAGATAGTCA
FabA	CCATTTCATTGGCGACCCG	CCGCAGCAGTGTAGATAACG

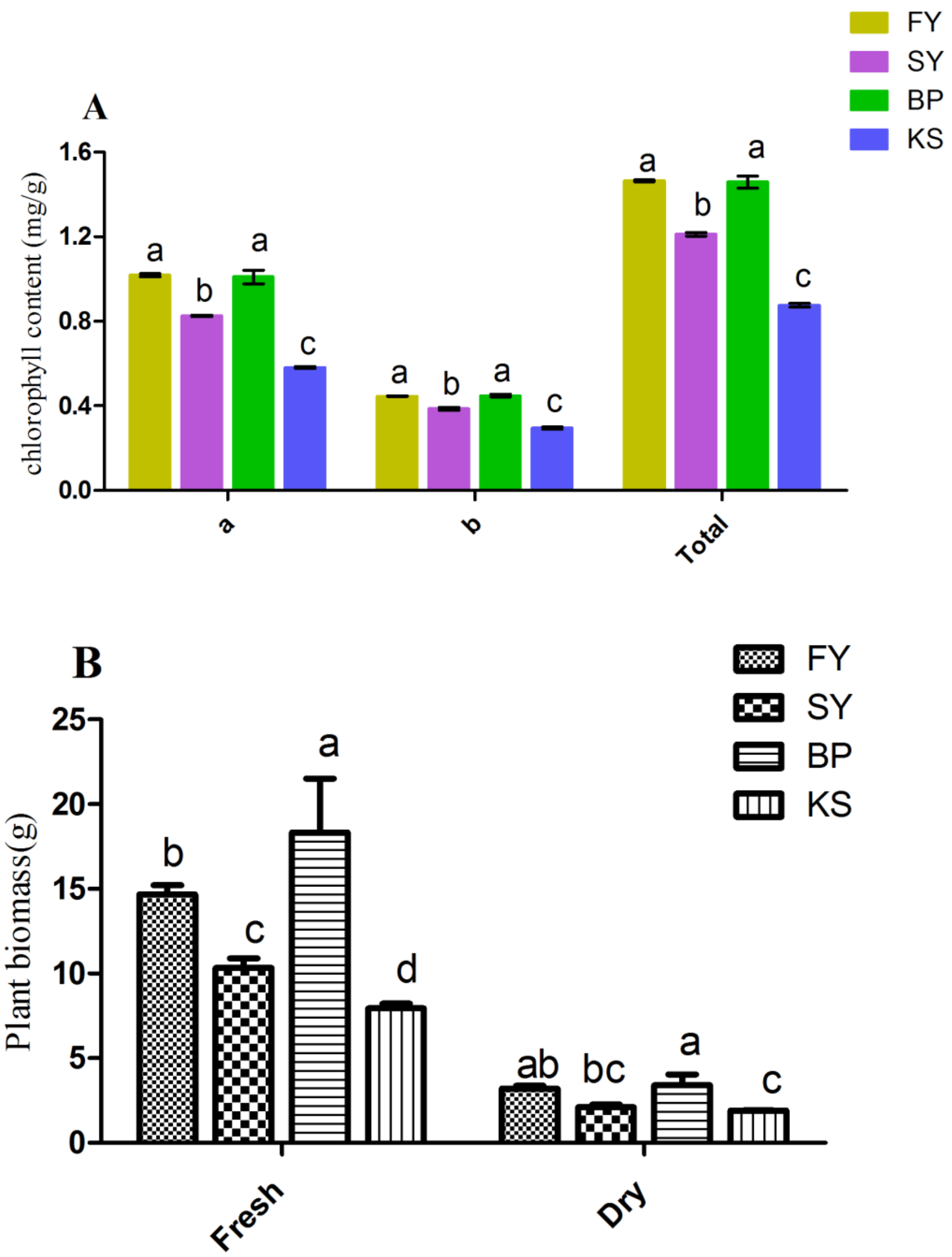

Figure S1. Chlorophyll content and plant biomass of *Radix pseudostellariae* under different treatments. FY represents the first cropping year, SY the second cropping year, BP the treatment of *Bacillus pumilus* in the second cropping year, and KS the treatment of *Kosakonia sacchari* in the first cropping year.

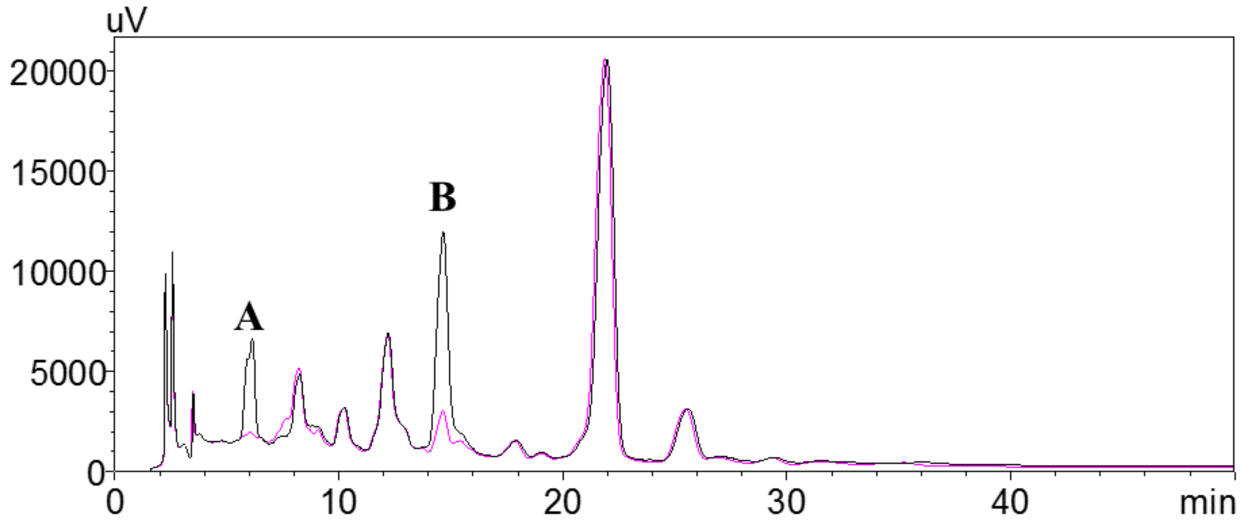

Figure S2 Chromatogram of phenolic compounds detected by high-performance liquid chromatography. Red chromatogram means the sample. Black chromatogram means the sample that was added to the standard compounds. A represents protocatechuic acid and B vanillin.

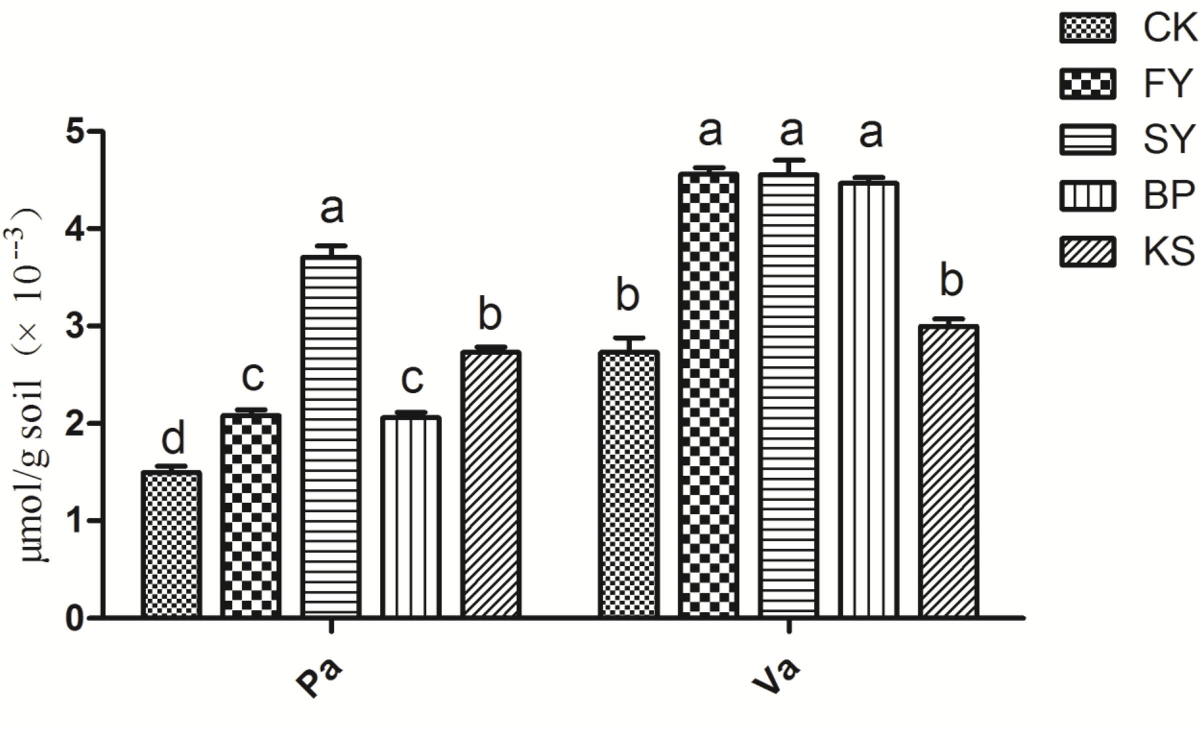

Figure S3 Phenolic contents in the rhizosphere soil of *Radix pseudostellariae* under different treatments.

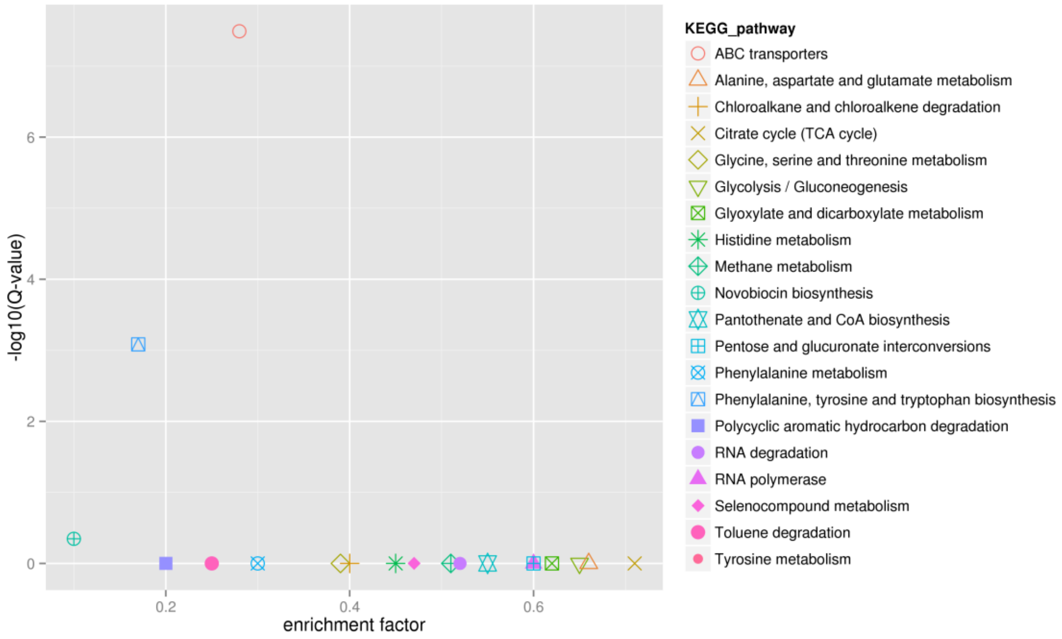

Figure S4 Top 20 KEGG pathway enrichment scatter diagram of the DEG between sample treatment (BP) and control (BPCK) in *Bacillus pumilus*.

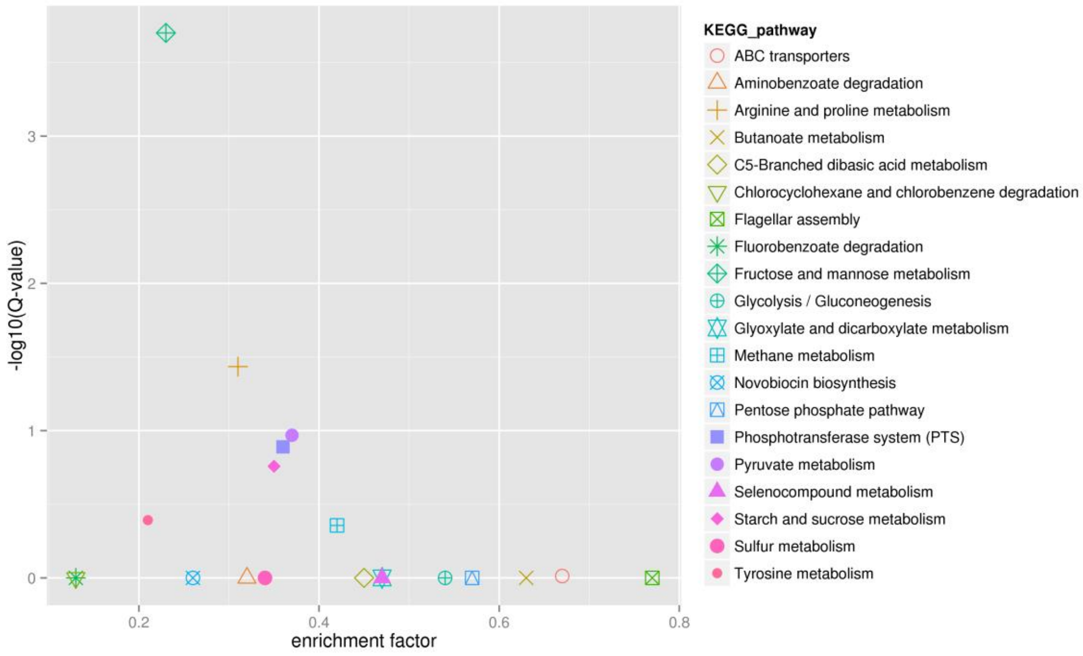

Figure S5. Top 20 KEGG pathway enrichment scatter diagram of the DEG between sample treatment (SM2) and control (SMCK2) in *Kosakonia sacchari*.

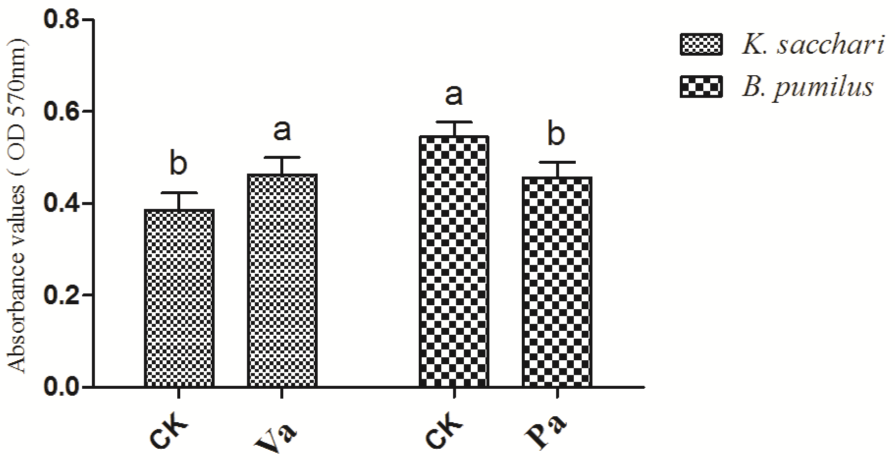

Figure S6 Effects of phenolic acids at a dose of 120 μmol/L on biofilm formation of bacteria. Pa represents protocatechuic acid and Va vanillin. Columns with different letters are statistically different (least significant difference test, $P < 0.05$).
